# Supplementary figures and images for: scGRN-Entropy: Inferring cell differentiation trajectories using single-cell data and gene regulation network-based transfer entropy
Source: PLoS Comput Biol. 2024 Nov 25;20(11):e1012638. doi: 10.1371/journal.pcbi.1012638 (PMC11627384; doi:10.1371/journal.pcbi.1012638)

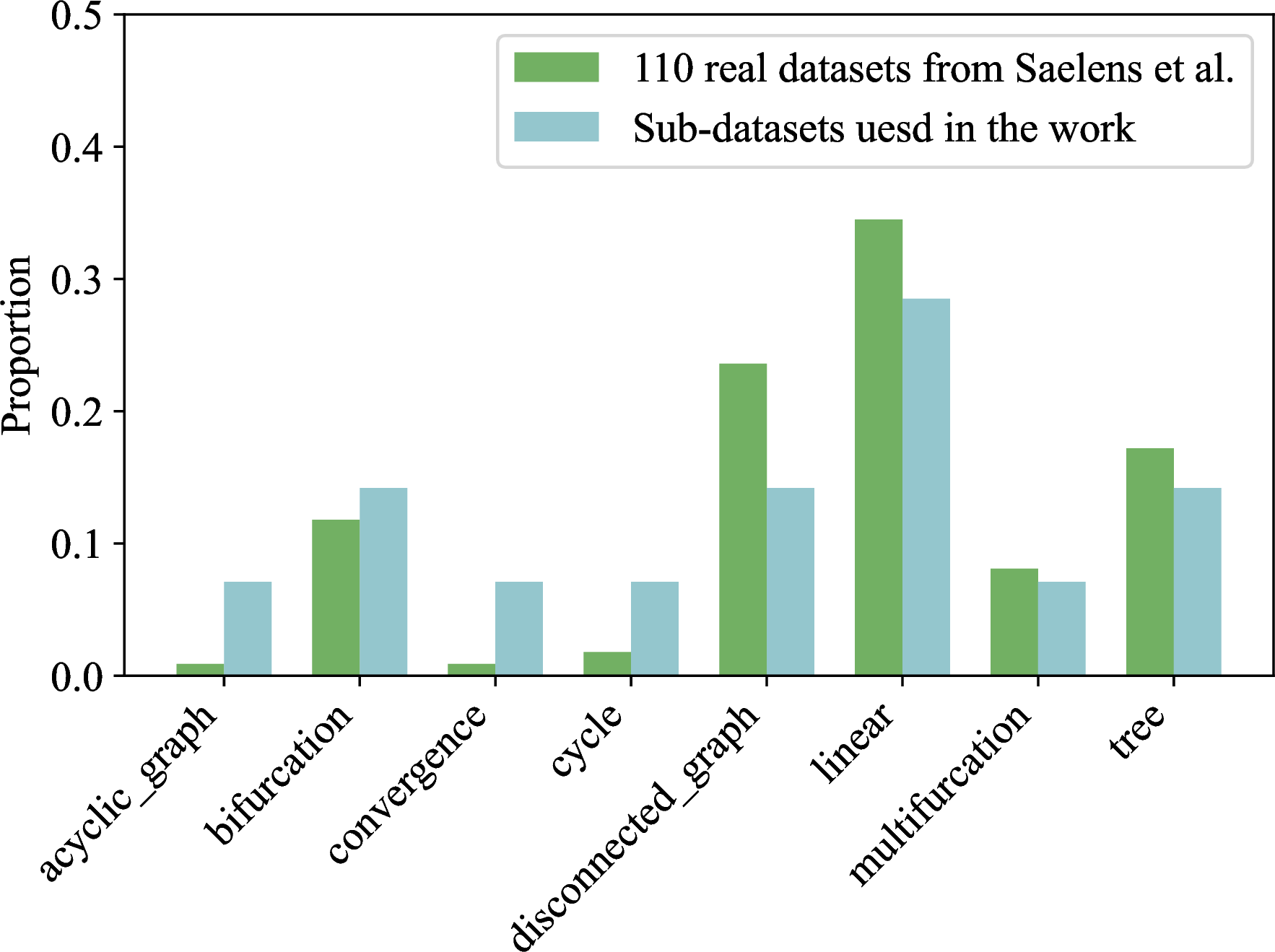

Supplement: S1 Fig — Since the acyclic_graph, convergence, and cycle trajectory types have very little data (the numbers are 1, 1, 2, respectively), our pro-portion on these trajectory types is significantly higher than the proportion of the original 110 real data sets. (TIF) [file pcbi.1012638.s001.tif]

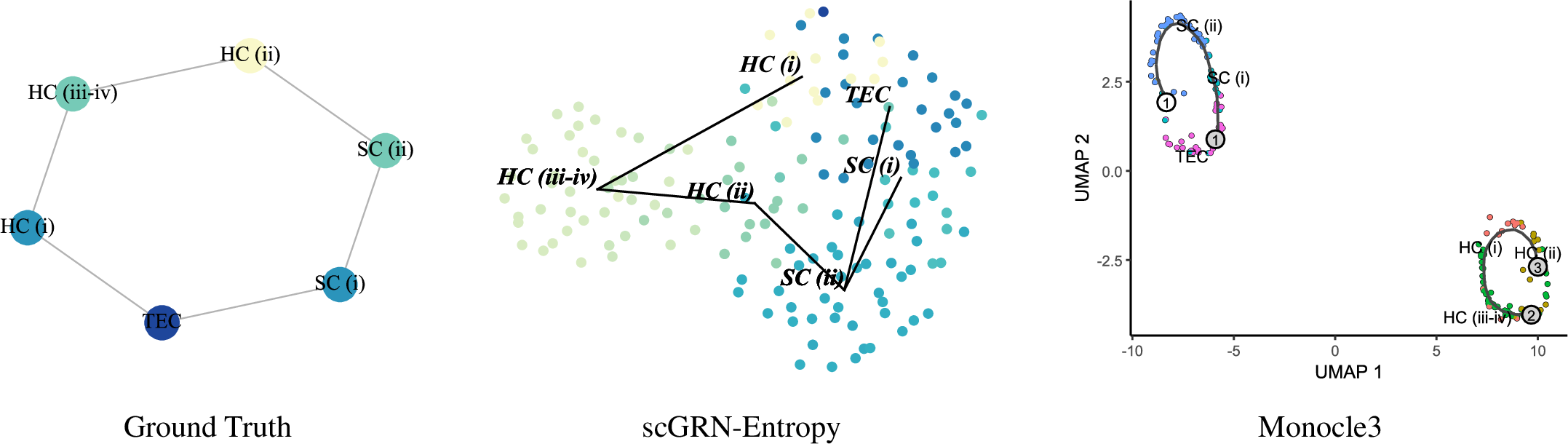

Supplement: S2 Fig — (TIF) [file pcbi.1012638.s002.tif]

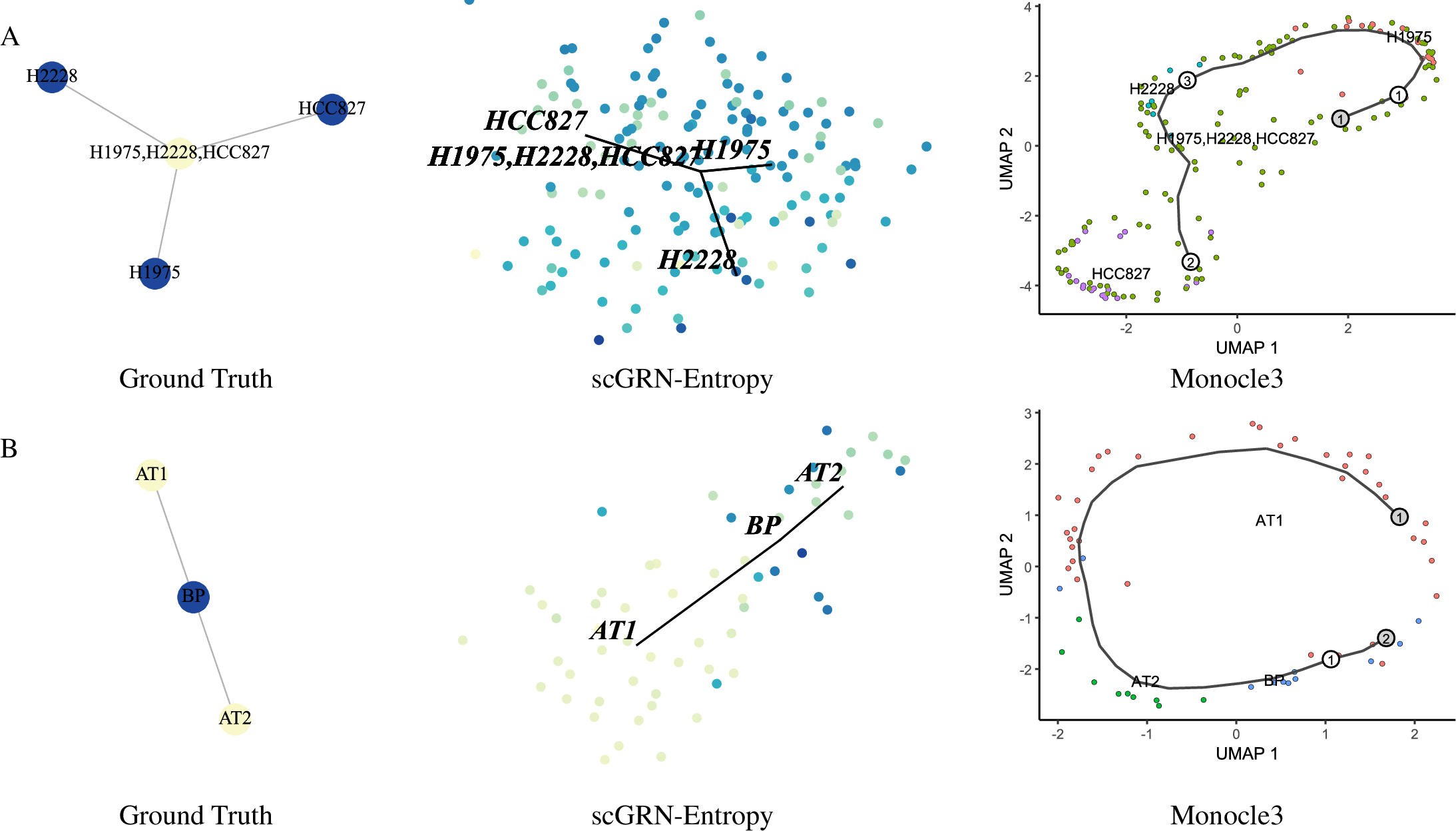

Supplement: S3 Fig — (TIF) [file pcbi.1012638.s003.tif]

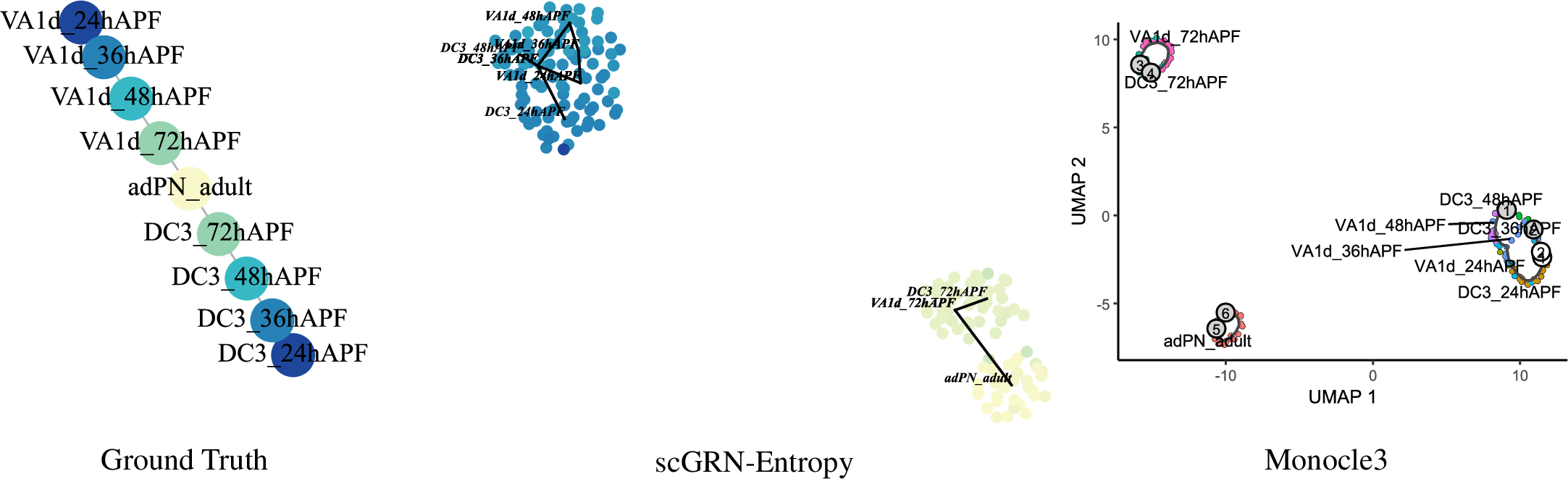

Supplement: S4 Fig — (TIF) [file pcbi.1012638.s004.tif]

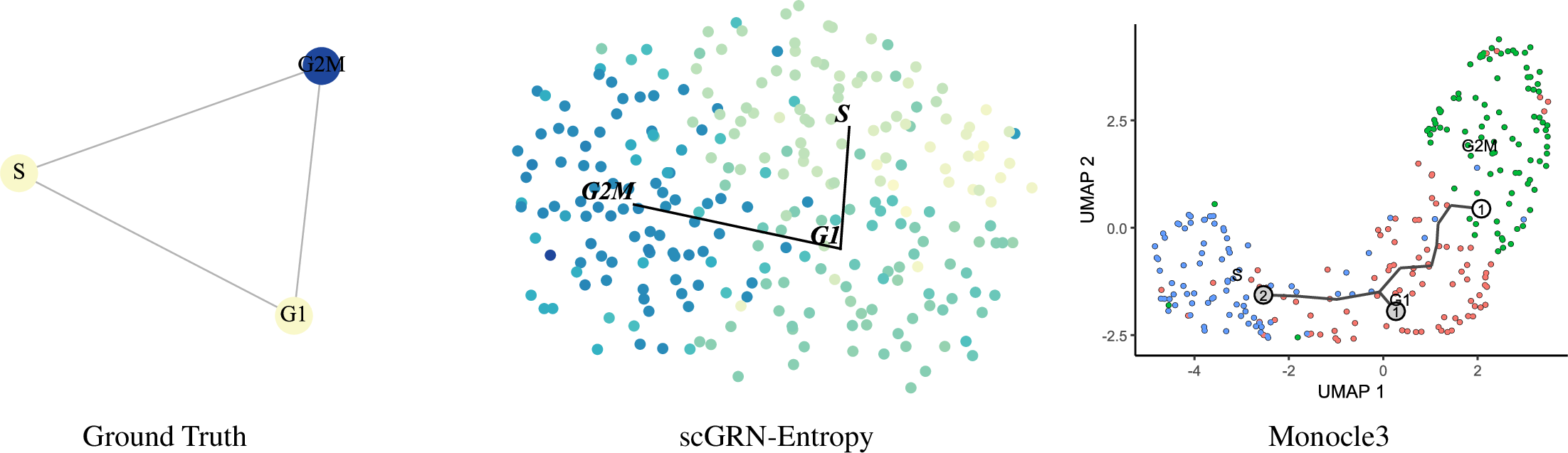

Supplement: S5 Fig — (TIF) [file pcbi.1012638.s005.tif]

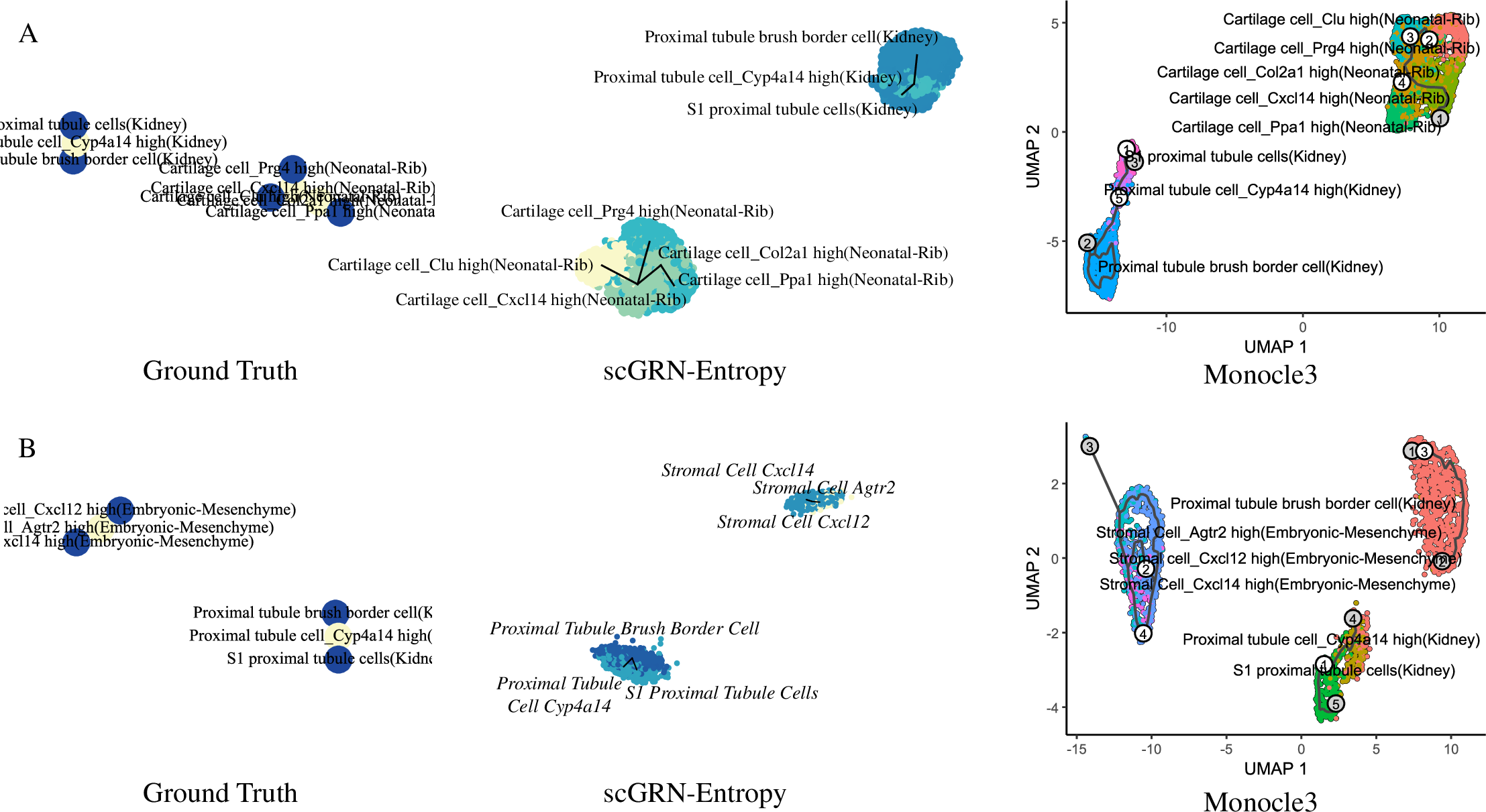

Supplement: S6 Fig — (TIF) [file pcbi.1012638.s006.tif]

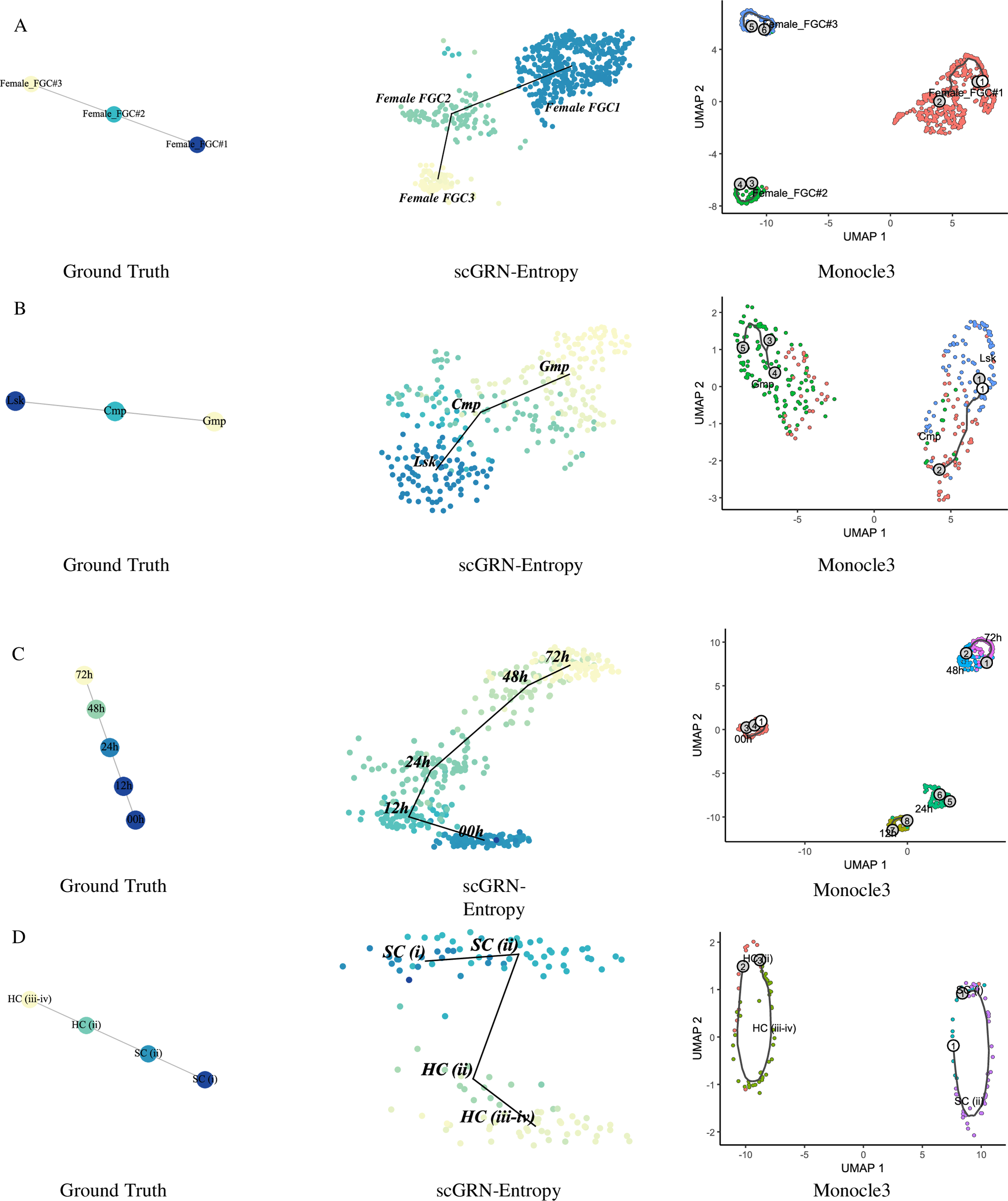

Supplement: S7 Fig — Comparisons of the differentiation trajectories inferred by scGRN-Entropy (middle) and Monocle3 (right) with the ground truth trajectory (left) for 4 linear type datasets: (A) the germline-human-female_li dataset, and (B) the hematopoiesis-gates_olsson dataset. (C) the mESC-differentiation_hayashi dataset, and (D) the neonatal-inner-ear-SC-HC_burns dataset. (TIF) [file pcbi.1012638.s007.tif]

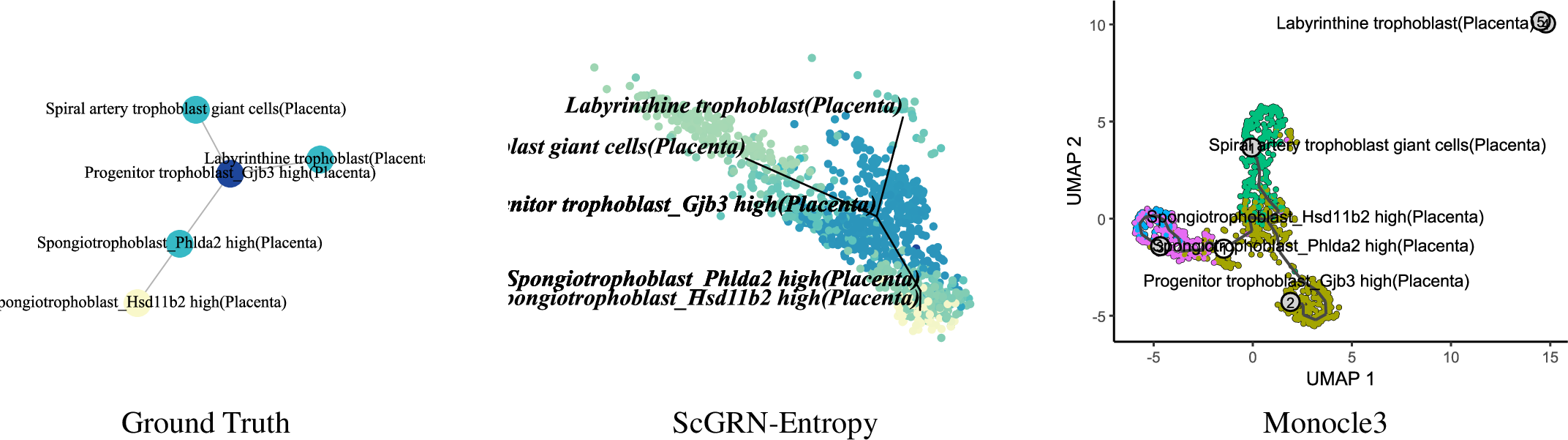

Supplement: S8 Fig — (TIF) [file pcbi.1012638.s008.tif]

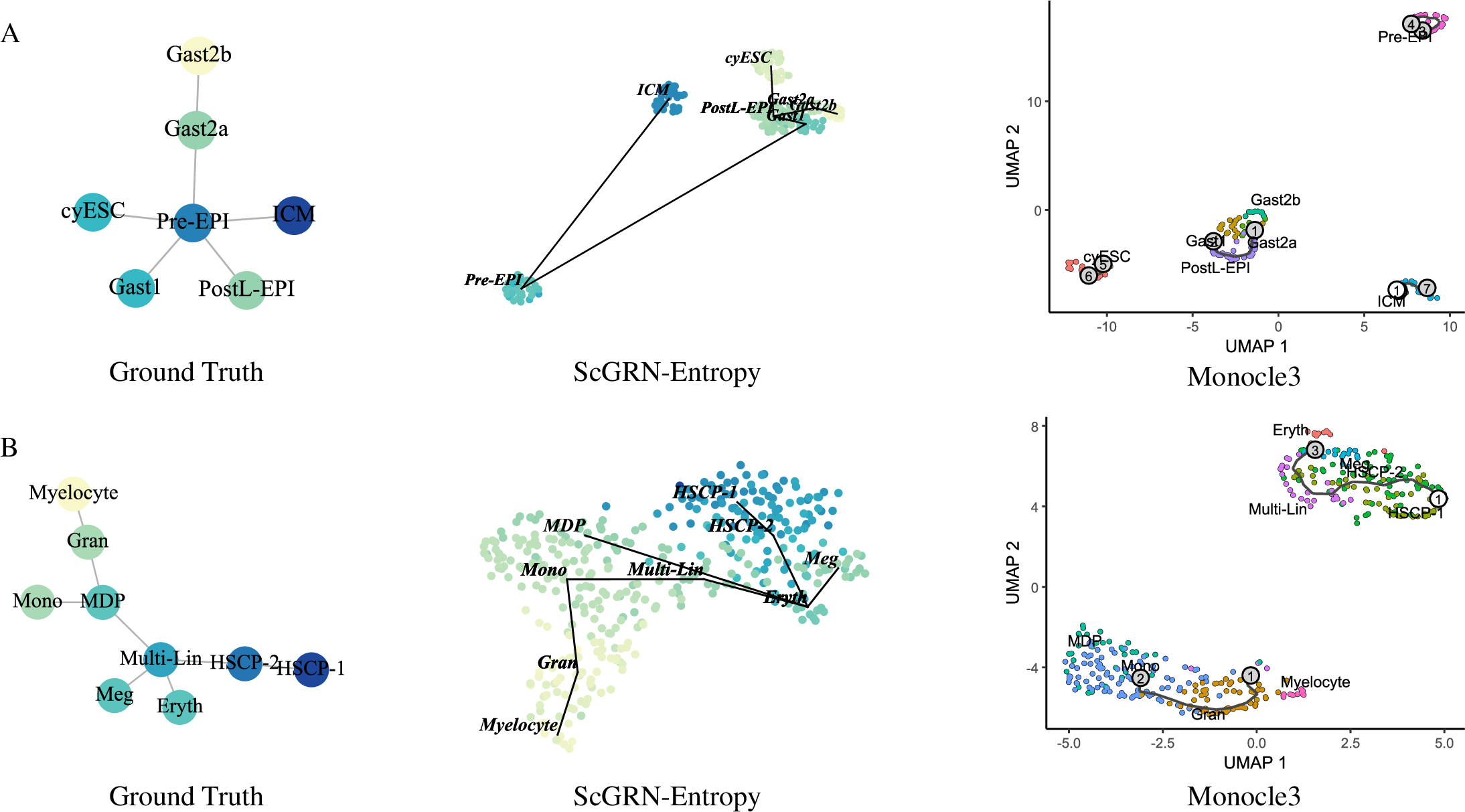

Supplement: S9 Fig — (TIF) [file pcbi.1012638.s009.tif]

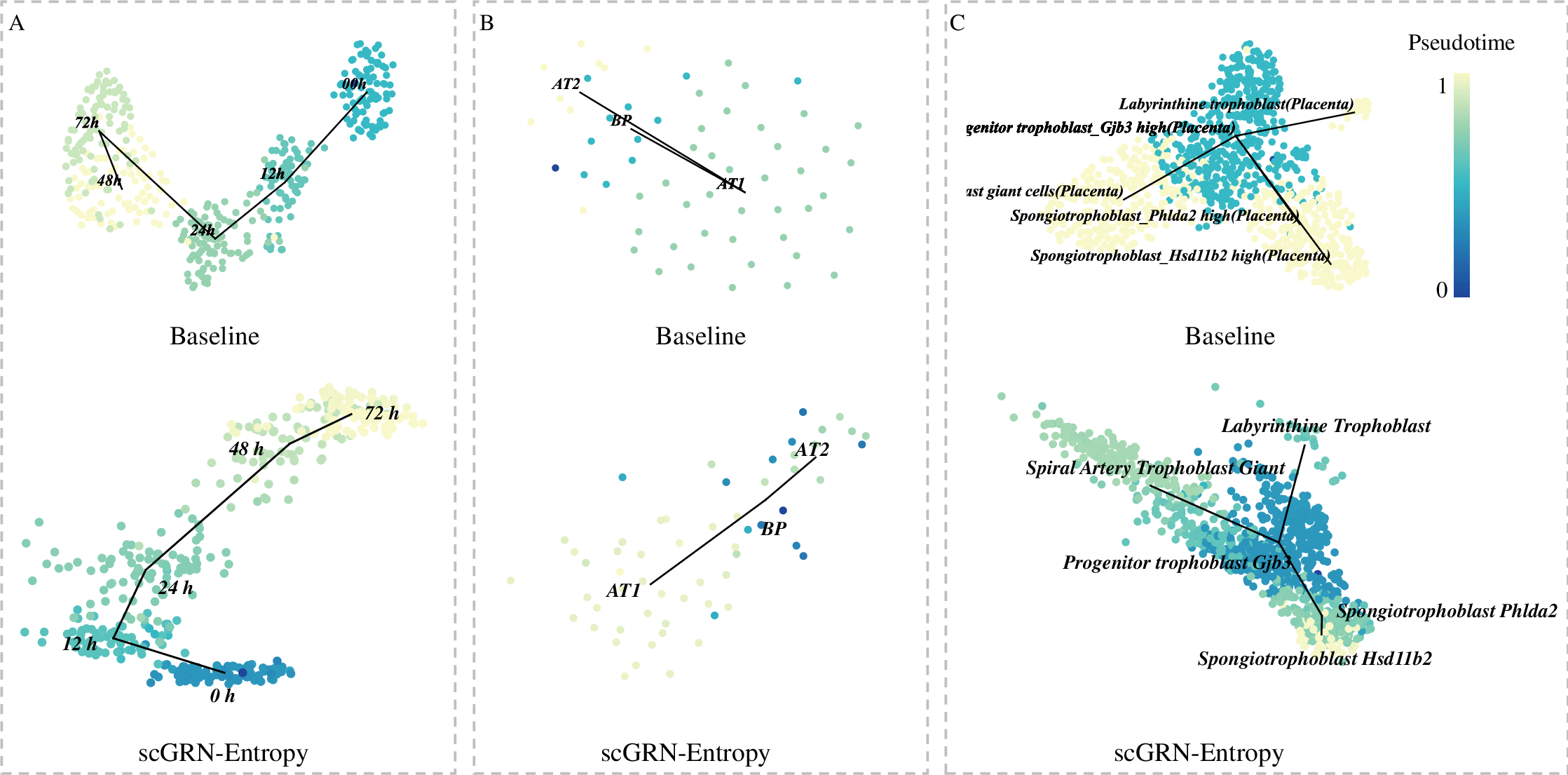

Supplement: S10 Fig — (A) For the mESC-differentiation_hayashi dataset, we obtained differentiation trajectories without considering the impact of GRN on the transition probability matrix, distance matrix, and pseudotime. We then compared these with trajectory inferences and pseudotime calculations con-sidering GRN’s influence. Without GRN, there were significant errors at 48h and 72h, where cells at 24h directly transitioned to 72h and then to 48h. However, with GRN, a continuous differentiation trajectory was achieved. (B) In the distal-lung-epithelium_treutlein dataset, we examined differ-entiation trajectories without and with GRN’s influence. BP cell clusters should differentiate into AT1 and AT2 clusters separately. Without GRN, BP first differentiated into AT1 and then into AT2. With GRN, pseudotime indicated a smaller time gap between AT1 and AT2 clusters. (C) For the placenta-trophoblast-differentiation_mca dataset, we analyzed differentiation trajectories without and with GRN’s effects. Without GRN, the progenitor trophoblast Gjb3 cluster directly differenti-ated into the spongiotrophoblast hsd11b2 cluster, skipping the spongiotrophoblast phlda2 cluster. Pseudotime without GRN had only initial and final points, while scGRN-Entropy provided a more continuous pseudotime. (TIF) [file pcbi.1012638.s010.tif]

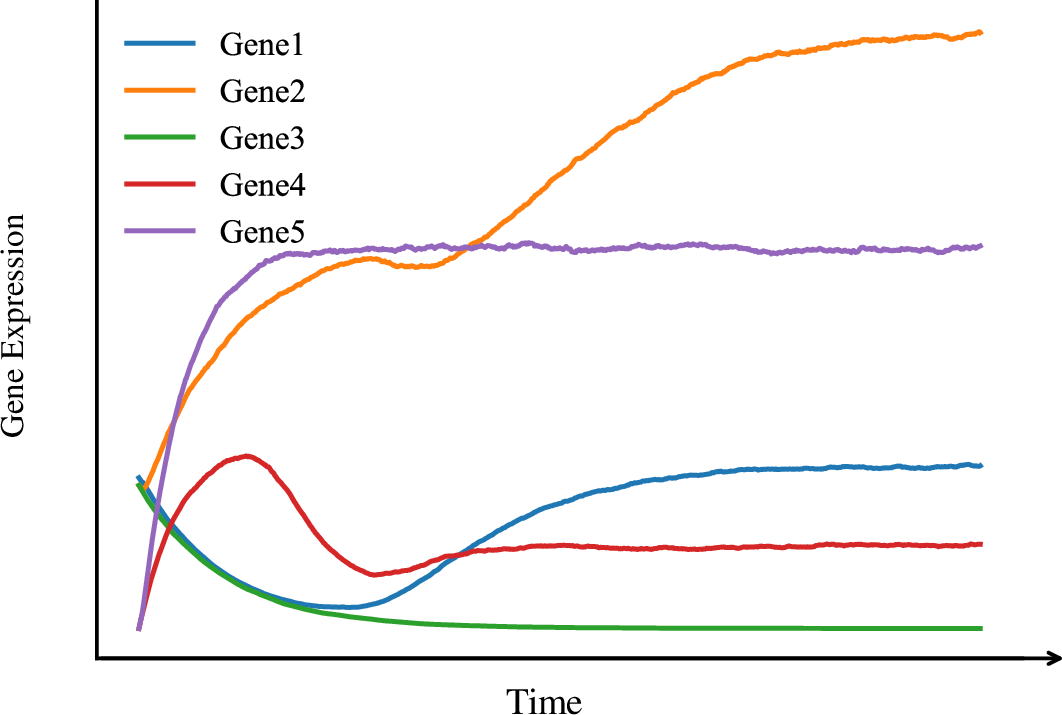

Supplement: S11 Fig — Gene5 reached a stable state first, while Gene2 reached a stable state only after all other genes had stabilized. Meanwhile, we assigned the gene expression values at each integer time point to individual cells. (TIF) [file pcbi.1012638.s011.tif]
