# Supplementary material for: scGRN-Entropy: Inferring cell differentiation trajectories using single-cell data and gene regulation network-based transfer entropy
Source: PLoS Comput Biol. 2024 Nov 25;20(11):e1012638. doi: 10.1371/journal.pcbi.1012638 (PMC11627384; doi:10.1371/journal.pcbi.1012638)
Supplement: Attachment — Submitted filename: Response_Letter to Reviewers.docx [file pcbi.1012638.s017.docx]

Dear reviewers,

Thank you very much for your help with reviewing our manuscript. We are resubmitting the revised version of our manuscript titled “scGRN-Entropy: Inferring Cell Differentiation Trajectories Using Single-Cell Data and Gene Regulation Network-Based Transfer Entropy”.

We are very grateful to you for the very thoughtful and helpful comments and suggestions. Your comments and suggestions improve our paper much more. In response, we have carefully revised the manuscript, with all changes highlighted in red color throughout the text. We address all the questions that you concerned and add the supporting information part in the revised version which can answer your comments much more clearly. We provide detailed responses to each of your comments and suggestions, addressing them point by point in the following attachment.

Thank you again! We are greatly appreciating your kind help on improving the quality of our manuscript. We hope the manuscript is now suitable for publication in *PLOS Computational Biology*.

Best wishes,

Sincerely

Ben-gong Zhang

[benyan1219@126.com](mailto:benyan1219@126.com)

School of Mathematical & Physical Sciences

Wuhan Textile University,Wuhan, 430200, China

**Response For Reviewer #1**

1. *In Table 1, eight diverse datasets of different trajectory types were included, selected* *from the Saelens et al. 2019 benchmarking paper. In Saelens et al.,* *110 real datasets and 229 synthetic datasets used for comparisons were released online, along with the codes. Could the authors provide a rationale for the selection of these specific eight datasets? For example, why were tree-based trajectories excluded from the study? Are these datasets chosen because they represent typical scenarios in the biological systems of interest, or were other factors considered in the selection?*

Thank you very much for your good questions. To validate our model, we randomly selected 8 datasets from the 110 real datasets in the Saelens et al. 2019 benchmarking paper. In line with your suggestion, we have now added datasets for each cell differentiation trajectory type, proportional to their number among the 110 real datasets (see Figure S1 in the Supplementary Materials). The final dataset comprises 14 cell groups, covering 8 distinct differentiation trajectory types (i.e., acyclic_graph, convergence, cycle, tree, bifurcation, linear, disconnected graph, and multifurcation). Detailed information of the datasets can be found in Table 1 as well as Table S1 in the Supplementary Materials. We have added the following sentences to address the issue.

“**Datasets and Evaluation metrics**

To assess the performance of scGRN-Entropy in inferring cell differentiation trajectories, we selected 14 real datasets from Ref. 14, where Saelens et al. developed a benchmark consisting of 110 real and 229 synthetic datasets for trajectory inference methods. Since the 110 real datasets include 8 different types of cell differentiation trajectories, we randomly selected a proportional number of datasets for each trajectory type (Fig S1). Additionally, the selected datasets covered four species (i.e. fly, human, macaque, and mouse) and six different sequencing technologies (Table S1). Each dataset includes both gene expression counts for each cell and the cellular groupings, along with the connections (directed edges) between these groups (e.g., group 1 → group 2 → group 3). These directed edges serve as the ground truth. Accordingly, accuracy, defined as the ratio of correctly predicted edges to the total number of edges, was used as a metric to evaluate predictive performance.” (page 8, lines 209-220)

2. *In Table 2,* *scGRN-Entropy was compared with other methods and showed accurate predictions on all 8 trajectories. In addition to trajectory, could the author also compare* *stability of predictions which is important for* *reproducibility in research? Also, adding a column specifying the trajectory type (bifurcation, linear, etc.) will enhance the interpretation of the results.*

Thank you very much for your valuable suggestions. Since the proposed method (i.e., scGRN-Entropy) does not rely on random factors, a given scRNA-seq data input will produce a consistent result. However, to improve the evaluation and reproducibility of predictions, we have replaced the previous True/False evaluation metric with trajectory prediction accuracy (see Methods and Table 1), and made the code of our method publicly available to allow readers to verify and utilize it. Additionally, we have included the trajectory type in Table 1 (previously Table 2 in the earlier version) to improve the interpretation of the results. Following sentences have been added/modified in the section of “Comparison with Existing Methods”.

“We compared our method with Slingshot [35], Monocle3 [36], and DBCTI [31] on these 14 datasets (Table 1 and Table S1), and all three methods were run using default script parameters. We used accuracy, defined as the true positive rate of the inferred trajectories relative to the ground truth, to evaluate the performance of all methods (Table 1). The average prediction accuracy of scGRN-Entropy is 84.1%, significantly outperforming Slingshot (78.4%), Monocle3 (58.9%), and DBCTI (48.8%). It should be noted that for 7 of the datasets, Slingshot failed to produce valid inference results, with the R script returning the error "the system computation is singular". These cases were marked as "None," and the average accuracy was calculated based on the other 7 datasets where valid predictions were returned. Since scGRN-Entropy, Slingshot, and Monocle3 rely on the minimum spanning tree algorithm for trajectory inference, they cannot accurately predict cycle-type trajectories (e.g., for acyclic graph and cycle types), although they can infer all connections except for those connected to the closed cell groups, such as the cell transition between the G2M and S2 phases in the cell-cycle_buettner dataset (Fig. S5). Accurately predicting complex tree-type trajectories remains challenging for existing methods. For example, in the epiblast-monkey nakamura and hematopoiesis-clusters olsson datasets, the prediction accuracy of scGRN-Entropy, Monocle3, and DBCTI is below 50%. Although scGRN-Entropy achieves an accuracy of 43% on the epiblast-monkey nakamura dataset, significantly higher than the 14% accuracy of the other two methods (Table 1), the inferred trajectory still deviates substantially from the experimental results; see Fig. S9 in the Supplementary Materials. Figs. S2-S9 in the Supplementary Materials also show the comparisons between the trajectories inferred by scGRN-Entropy and Monocle3 and the ground truth across the 14 datasets, suggesting that scGRN-Entropy provides more accurate predictions of cell differentiation trajectories, especially for linear types.” (page 10-11, lines 283-307)

3. *The uniqueness of* *scGRN-Entropy lies in its incorporation of cell distance in the GRN space. In Fig 2c, the author showed the decrease in performance when removing GRN information. Could the authors extend this analysis to other non-linear* *trajectory types? It will provide deeper insights into the effectiveness of GRN integration.*

Thank you very much for your good suggestion. To address the issue, we have added the following sentences.

“To demonstrate the importance of incorporating dynamic cell relationships, we also tested the scGRN-Entropy method without considering the dynamic cell relationships obtained from the GRN space (i.e., only using the cell relationship matrix in PCA space, treated as a baseline) on both linear and non-linear trajectory types. As shown in Fig. S10 in the Supplementary Materials, for mESC-differentiation_hayashi (linear type), the baseline model predicted an evolution direction opposite to the experiments at 48h and 72h. For distal-lung-epithelium_treutlein (bifurcation type) dataset, the baseline predictions incorrectly inferred the direction of terminal differentiation path, suggesting that BP first differentiates into AT1 and then into AT2. For the multifurcation-type dataset splacenta-trophoblast-differentiation_mca, the predicted progenitor trophoblast Gjb3 cluster directly differentiated into the spongiotrophoblast hsd11b2 cluster, bypassing the spongiotrophoblast phlda2 cluster. In contrast, for all the three datasets, the scGRN-Entropy method accurately predicted the differentiation trajectories, fully aligning with experimental results (i.e., 100% accuracy; see Table 1). Moreover, compared to the baseline results, scGRN-Entropy better distinguished the sequential order of differentiation among individual cells in terms of pseudotime (Fig. S10 in the Supplementary Materials). This demonstrates that the inclusion of GRN information significantly improved prediction accuracy across both linear and non-linear trajectory types, suggesting that GRNs may serve as an intrinsic driving force of cell differentiation.” (page 11, lines 308-327)

4. *For the gene set enrichment analysis, are the p-values reported raw values or multiple testing corrected? Also, in the KEGG enrichment analysis, despite selecting genes from the top/bottom 0.05 quantiles, the reported pathways are enriched by fewer than five genes. The enrichR package offers many libraries of pathways, is there any other pathway that showed more significant enrichment?*

Thank you for your insightful question. In Figure 5, the p-values reported are raw values. However, we agree that when performing multiple gene set tests, using adjusted p-values can effectively reduce the false positive rate. Therefore, we have updated our results to include a detailed report of the gene set enrichment analysis with both raw and adjusted p-values; see Table S2-S4 in the Supplementary Materials. To address the issue, we have added/modified the following sentences.

“Furthermore, we performed enrichment analysis on this dataset to explore how accurate trajectory inference contributes to understanding the roles of these genes in cellular signal transduction, metabolism, and other complex biological processes. First, we calculated the correlation between each gene's expression level across all cells and the inferred pseudotime. Using a significance threshold of 0.05, we selected genes that showed a significant positive or negative correlation with pseudotime. Enrichment analysis was then conducted on these two gene sets separately using EnrichR [53,54] (see Fig. 4 and Tables S2-S4 in the Supplementary Materials). Among the genes positively correlated with pseudotime, KEGG analysis [55-58] revealed pathways related to the development of female reproductive organs, such as Progesterone-mediated oocyte maturation (Homo sapiens hsa04914), Oocyte meiosis (Homo sapiens hsa04114), Cell cycle (Homo sapiens hsa04110), and FoxO signaling pathway (Homo sapiens hsa04068) (Fig 4A). As shown in Fig 4B, GO analysis of the positively correlated genes identified biological processes related to gonadal development and the cell cycle (with p-values/adjusted p-values < 0.001). These processes include homologous chromosome pairing at meiosis (GO:0007129), piRNA metabolic process (GO:0034587), homologous chromosome segregation (GO:0045143), meiosis I (GO:0007127), meiotic DNA double-strand break formation (GO:0042138), meiotic sister chromatid cohesion (GO:0051177), and ncRNA metabolic process (GO:0034660); see Table S3 in the Supplementary Materials. In the top 10 pathways with the smallest p-values, there are many molecular function pathways related to cell growth and development. For genes negatively correlated with pseudotime, KEGG analysis only identified the Focal adhesion (Homo sapiens hsa04510) pathway as being related to reproductive development, with no statistically significant pathways related to the cell cycle (Fig 4C). GO analysis confirmed significant associations with apoptotic processes, such as the intrinsic apoptotic signaling pathway mediated by p53 (GO:0072332) and the defense response to viruses (GO:0051607); see Fig. 4D and Table S4 in the Supplementary Materials. Unfortunately, KEGG analysis for both positively and negatively correlated genes did not get small enough p-value, which may be due to the relatively small number of genes selected (<100).” (page 12-13, lines 343-373)

5. *Table 3 is not referenced anywhere in the manuscript. Also, both row and column labels are missing.*

Thank you very much for your kind reminder. We apologize for this oversight. We have now improved Table 2 (previously Table 3 in the earlier version) and updated the corresponding reference in the main text.

“The stoichiometric matrix and propensity function of the chemical reaction (Table 2) are as follows [22].” (page 13, lines 377-378)

6. *Making the codes of scGRN-Entropy publicly available would allow other researchers to validate the findings. The code and data* *availability link (https://zenodo.org/records/1443566%5C#.Y3q1fnbMKUl) isn’t working.*

Thank you for your good suggestion and kind reminder. We have now made the codes of scGRN-Entropy publicly available and modified the data availability link in the revised version. It does works now.

“All datasets can be downloaded via https://zenodo.org/records/1443566. All code about scGRN-Entropy can be download from https://github.com/SuunRui/scGRN-Entropy.git.” (page 16, lines 452-454)

7. *Fig 1B was introduced before Fig 1A*.

Thank you for your kind reminder. We have corrected it now.

“To construct the gene regulatory network for each cell (Fig. 1A(II)), we first perform gene pooling [32], which involves dimensionality reduction by clustering genes based on their correlations across all cells.” (page 3, lines 92-93)

8. *In Methods- Datasets, it’s unclear why some datasets are categorized as gold while others as silver standard.*

Thank you for your thoughtful reminder. The dataset standard was defined by Saelens et al. (Nature Biotechnology. 2019; 37(5):547–554), where datasets were classified as gold or silver standard based on how the labels were determined: silver standard labels were assigned by experts using expression data, while gold standard labels were based on external information such as FACS or sample origin. In scGRN-Entropy, the cellular groupings are directly used as input, without distinguishing between gold or silver standards; thus, we have removed any references to these classifications.

9. *In the Fig 3 caption, the panel labels were not discussed in alphabetical order. This might be due to the shape and space constraints of the panels, but reordering them will improve clarity and readability*.

Thank you very much for your good suggestion. We have now revised the caption of Figure 3. (page 7)

10. *How many supergenes were chosen in each of the validation dataset?*

Thank you for your question. We have now included the number of supergenes for each dataset in Table 1.

11. *Fig 3F, the size of the dots is different from the other panels*. *Fig 4B, the labels for FGC1, FGC2, FGC3 are missing. Fig 2C was cited at the very beginning of the Results, while Fig 2A and 2B were introduced in the last subsection of Results.*

We appreciate your careful review and suggestions. We have made the following adjustments: (1) The dot sizes in Figure 3F have been adjusted to be consistent with the other panels. (2) The labels for FGC1, FGC2, and FGC3 have been added to Figure 4B. (3) The citation order of all the figures/tables has been carefully reviewed and revised to ensure logical flow.

**Response For Reviewer #2**

1. *The manuscript lacks a clear definition of ground truth for benchmarking. It is unclear how to determine true/false in this context. what means “**completely correct” and “not completely correct”? Is it including miss ordering of even 1 cell considered as False? A quantitative metric is essential for objectively evaluating the performance of the proposed method compared to existing approaches.*

Thank you very much for your good questions and suggestions. To address these issues, we have added a clear definition of the ground truth and replaced the "completely/non-completely correct" classification with prediction accuracy to quantitatively and objectively assess the performance of the methods (see Methods and Table 1). Following sentences are added in the manuscript.

“We compared our method with Slingshot [35], Monocle3 [36], and DBCTI [31] on these 14 datasets (Table 1 and Table S1), and all three methods were run using default script parameters. We used accuracy, defined as the true positive rate of the inferred trajectories relative to the ground truth, to evaluate the performance of all methods (Table 1). The average prediction accuracy of scGRN-Entropy is 84.1%, significantly outperforming Slingshot (78.4%), Monocle3 (58.9%), and DBCTI (48.8%). It should be noted that for 7 of the datasets, Slingshot failed to produce valid inference results, with the R script returning the error "the system computation is singular". These cases were marked as "None," and the average accuracy was calculated based on the other 7 datasets where valid predictions were returned. Since scGRN-Entropy, Slingshot, and Monocle3 rely on the minimum spanning tree algorithm for trajectory inference, they cannot accurately predict cycle-type trajectories (e.g., for acyclic graph and cycle types), although they can infer all connections except for those connected to the closed cell groups, such as the cell transition between the G2M and S2 phases in the cell-cycle_buettner dataset (Fig. S5). Accurately predicting complex tree-type trajectories remains challenging for existing methods. For example, in the epiblast-monkey nakamura and hematopoiesis-clusters olsson datasets, the prediction accuracy of scGRN-Entropy, Monocle3, and DBCTI is below 50%. Although scGRN-Entropy achieves an accuracy of 43% on the epiblast-monkey nakamura dataset, significantly higher than the 14% accuracy of the other two methods (Table 1), the inferred trajectory still deviates substantially from the experimental results; see Fig. S9 in the Supplementary Materials. Figs. S2-S9 in the Supplementary Materials also show the comparisons between the trajectories inferred by scGRN-Entropy and Monocle3 and the ground truth across the 14 datasets, suggesting that scGRN-Entropy provides more accurate predictions of cell differentiation trajectories, especially for linear types.” (page 10-11, lines 283-307)

2. *Necessity of GRN Space Similarity. The authors should explore the impact of using only static similarity (without GRN space similarity) on the inferred pseudotime, KNN graph, and trajectories. This would help to elucidate the contribution of GRN information to the method's performance and identify potential limitations of relying solely on GRN-based similarity*.

Thank you very much for your good suggestion. To address the issue, we have added the following sentences.

“To demonstrate the importance of incorporating dynamic cell relationships, we also tested the scGRN-Entropy method without considering the dynamic cell relationships obtained from the GRN space (i.e., only using the cell relationship matrix in PCA space, treated as a baseline) on both linear and non-linear trajectory types. As shown in Fig. S10 in the Supplementary Materials, for mESC-differentiation_hayashi (linear type), the baseline model predicted an evolution direction opposite to the experiments at 48h and 72h. For distal-lung-epithelium_treutlein (bifurcation type) dataset, the baseline predictions incorrectly inferred the direction of terminal differentiation path, suggesting that BP first differentiates into AT1 and then into AT2. For the multifurcation-type dataset splacenta-trophoblast-differentiation_mca, the predicted progenitor trophoblast Gjb3 cluster directly differentiated into the spongiotrophoblast hsd11b2 cluster, bypassing the spongiotrophoblast phlda2 cluster. In contrast, for all the three datasets, the scGRN-Entropy method accurately predicted the differentiation trajectories, fully aligning with experimental results (i.e., 100% accuracy; see Table 1). Moreover, compared to the baseline results, scGRN-Entropy better distinguished the sequential order of differentiation among individual cells in terms of pseudotime (Fig. S10 in the Supplementary Materials). This demonstrates that the inclusion of GRN information significantly improved prediction accuracy across both linear and non-linear trajectory types, suggesting that GRNs may serve as an intrinsic driving force of cell differentiation.” (page 11, lines 308-327)

3. *A more compelling application is needed. While the enrichment results obtained from the analysis in case study are interesting, it is important to demonstrate the unique advantages of the proposed method compared to existing approaches. The authors should discuss how their method can lead to novel discoveries or more reasonable results that are not achievable with current techniques.*

Thank you very much for your kind comment and good suggestions. We agree that emphasizing the unique advantages of scGRN-Entropy is crucial. Unlike existing approaches, scGRN-Entropy improves inference accuracy by incorporating dynamic changes in gene regulatory networks (GRN). We tested scGRN-Entropy across 14 datasets representing eight different types of differentiation trajectories and compared it with several state-of-art methods. The average prediction accuracy of scGRN-Entropy is 84.1%, significantly outperforming Slingshot (78.4%), Monocle3 (58.9%), and DBCTI (48.8%). Moreover, we conducted additional tests using scGRN-Entropy without considering the dynamic cell relationships from the GRN space (i.e., using only the cell relationship matrix in PCA space, treated as a baseline). These tests, on both linear and non-linear trajectory types, further confirmed that incorporating GRN information significantly improved prediction accuracy. This finding suggests that GRNs may play an intrinsic role in driving cell differentiation. To address the issue, we have added/modified the following sentences.

“We validated the effectiveness of our method on 14 real-world datasets covering various trajectory types, successfully obtaining continuous cell differentiation trajectories and pseudotime. Although scGRN-Entropy has certain limitations when inferring circular and complex tree-type trajectories (e.g., it cannot form closed trajectories), it achieved an average prediction accuracy of 84.1%, which is significantly higher than that of current state-of-the-art methods.” (page 14-15, lines 395-403)

“The dynamic changes in gene expression during cell differentiation are crucial for understanding cellular and organismal life processes [60,61]. Our work not only introduces a new trajectory inference method but also highlights the contribution of dynamic changes in the GRN over time to cell differentiation. Comparisons with the baseline results demonstrate that the inclusion of GRN information significantly enhances the accuracy of scGRN-Entropy in predicting differentiation trajectories and provides a more detailed depiction of the order of cell differentiation. This suggests that dynamic gene regulation may be one of the primary driving forces of cell differentiation. Unfortunately, the current method is still limited in its ability to fully resolve the complex regulatory networks involving thousands of genes that are simultaneously expressed in a cell at any given moment [62,63]. Nevertheless, scGRN-Entropy reduces the dimensionality based on gene expression relationships, aiming to retain the interpretability of the principal components while also preserving as much of the gene regulatory network's integrity as possible.” (page 15, lines 404-417)
